# Endogenously-Produced Hyaluronan and Its Potential to Regulate the Development of Peritoneal Adhesions

**DOI:** 10.3390/biom12010045

**Published:** 2021-12-29

**Authors:** Anna Kocurkova, Kristina Nesporova, Miriam Sandanusova, Michaela Kerberova, Katerina Lehka, Vladimir Velebny, Lukas Kubala, Gabriela Ambrozova

**Affiliations:** 1 Institute of Biophysics, Academy of Sciences of the Czech Republic, 612 65 Brno, Czech Republic; 423464@mail.muni.cz (A.K.); 451779@mail.muni.cz (M.S.); 459912@mail.muni.cz (M.K.); kubalal@ibp.cz (L.K.); 2 Institute of Experimental Biology, Faculty of Science, Masaryk University, 611 37 Brno, Czech Republic; 3 International Clinical Research Center, St. Anne’s University Hospital, 656 91 Brno, Czech Republic; 4 Contipro a.s., Dolní Dobrouč 401, 561 02 Dolní Dobrouč, Czech Republic; Kristina.Nesporova@contipro.com (K.N.); Katerina.Lehka@contipro.com (K.L.); Vladimir.Velebny@contipro.com (V.V.)

**Keywords:** peritoneal adhesion, fibrosis, hyaluronan, mesothelial cell, metabolism, inflammation, mesothelial-to-mesenchymal transition

## Abstract

Formation of peritoneal adhesions (PA) is one of the major complications following intra-abdominal surgery. It is primarily caused by activation of the mesothelial layer and underlying tissues in the peritoneal membrane resulting in the transition of mesothelial cells (MCs) and fibroblasts to a pro-fibrotic phenotype. Pro-fibrotic transition of MCs—mesothelial-to-mesenchymal transition (MMT), and fibroblasts activation to myofibroblasts are interconnected to changes in cellular metabolism and culminate in the deposition of extracellular matrix (ECM) in the form of fibrotic tissue between injured sides in the abdominal cavity. However, ECM is not only a mechanical scaffold of the newly synthetized tissue but reciprocally affects fibrosis development. Hyaluronan (HA), an important component of ECM, is a non-sulfated glycosaminoglycan consisting of N-acetyl-D-glucosamine (GlcNAc) and D-glucuronic acid (GlcUA) that can affect the majority of processes involved in PA formation. This review considers the role of endogenously produced HA in the context of different fibrosis-related pathologies and its overlap in the development of PA.

## 1. Introduction

Peritoneal adhesions (PA) represent one of the major complications following intraabdominal and pelvic surgery leading to symptoms such as abdominal pain, bowel obstruction and infertility. The molecular mechanism of this phenomenon is not sufficiently understood, however, it is very frequent (incidence up to 90%) and well described in clinical practice even after laparoscopic surgeries [1,2]. Frequently, PA are formed randomly among organs distant from the site of the mechanical disruption. Thus, their formation is currently recognized to be driven by a complex process where not only a disruption of the mesothelial layer and its underlying structures in the peritoneal membrane but also hypoxia, inflammation, and deregulated coagulation/fibrinolysis in the peritoneal cavity participate. During the initial phases, pro-fibrotic transition of mesothelial cells (MCs), forming an inside monolayer of the peritoneal membrane, and a fibroblast-myofibroblast switch of fibroblasts from underlying tissues plays a central role in the alterations of the peritoneal membrane leading to fibrosis [3,4]. The mesothelial-to-mesenchymal transition (MMT) consists of gradual loss of epithelial characteristics and the acquisition of myofibroblast-like phenotype. Subsequently, deposition of extracellular matrix (ECM) by myofibroblasts is induced and fibrotic tissue is formed among visceral organs [3,5].

Usage of anti-adhesive barriers, designed to keep opposing surfaces separated at the site of the lesion, is currently a highly expanding strategy to inhibit fibrotic tissue formation in the peritoneum. A concise summary of available commercial products designed as mechanical barriers to prevent fibrosis development in various tissues is currently provided by Capella-Monsonis et al. [6]. Hyaluronan (HA) polymers are one of the crucial compounds of most of the developed solid and semisolid barriers. Currently, there is satisfactorily reviewed that exogenously applied HA can affect the development of PA [1,6]. However, there is a significant lack of information concerning the regulatory properties of HA produced by cells involved in the development of PA or peritoneal fibrosis in general. Similarly, the effect of HA on the metabolism of cells participating in ECM deposition as well as the role of enzymes responsible for HA synthesis or degradation and HA receptors is poorly defined in the context of peritoneal fibrosis. Therefore, this review aims to describe the anticipated effects of endogenously produced HA on fundamental mechanisms responsible for the development of PA inspired by different relevant fibrosis-related pathologies. 

## 2. Regulatory Role of ECM Components in Fibrosis

ECM is produced by cells into their surroundings and represents the non-cellular part of all organs and tissues. It is formed by plenty of different proteins, glycoproteins, proteoglycans, and glycosaminoglycans. Moreover, cytokines, growth factors, and ECM-remodeling enzymes are present in this network of macromolecules [7]. The precise composition of ECM and ratio of the mentioned macromolecules are specific for different tissues and influence their structure and properties. ECM components serve not only as a mechanical scaffold for cells but also as regulatory molecules affecting cell growth, differentiation, migration, metabolism, and survival through interaction with specific receptors such as integrins, syndecans, and discoidin domain receptors [8].

In general, ECM is a highly dynamic structure that is continuously being rebuilt and remodeled by both anabolic and catabolic enzymes. This process of remodeling is strictly controlled and regulated, and failure of this control leads to the development of various pathologies. The pathophysiological condition characterized by excessive production and deposition of ECM, usually resulting from a previous injury, is known as fibrosis [9]. The changing ratio of ECM components to cell mass can be an accompanying effect modulating ECM properties. Crosslinking of various proteins by tissue transglutaminase 2 ensures its stiffness and higher stability [10]. Mechanosensitive Hippo pathway is activated due to increased stiffness, and deposition of ECM is upregulated as a result of positive feedback loop [11]. Thus, the alternations of ECM during fibrotic diseases are not only consequences but also contribute to fibrosis development. For example, decellularized ECM derived from a patient with idiopathic pulmonary fibrosis is able to switch fibroblasts to activated myofibroblasts [11].

Concerning the particular ECM components and their potential to regulate the cell phenotype, the most abundant protein and one of the most crucial components of ECM is collagen, mainly synthetized by fibroblasts. Its main function is to provide tensile strength to tissues [12]. However, proteolytic cleavage of collagen leads to the production of bioactive fragments promoting fibrosis by stimulation of fibroblasts [13]. Elastin, another highly abundant ECM protein with stable fibers participating in ECM composition, creates a large network that is responsible for the flexibility of tissues. However, in fibrotic livers, an increased ECM stiffness was reported due to a two-fold increase in elastin content and its crosslinking while the downregulation of genes responsible for elastin cross-linking leads to fibrosis alleviation [14]. Fibronectin, a glycoprotein found both soluble in plasma and insoluble in cell-associated form, has an important role in embryonic development and tissue regeneration. Moreover, as well as in the case of collagens, fragments of fibronectin are supposed to be fibrogenic [15]. 

Except for the described proteins and glycoproteins, proteoglycans are crucial parts of healthy and pathologically modified ECM. They interact with a majority of molecules of the ECM network (pronounced in the hyalectan family of proteoglycans) that gives them the function of ECM organizers and scaffolds. A typical example is versican, a large extracellular hyalectan, which binds lectin by its C-terminal domain, and the N-terminal domain interacts with HA creating an information-rich network of glycans and proteins [16]. However, proteoglycans also significantly affect cell migration, adhesion, proliferation, and differentiation.

## 3. Hyaluronan (Hyaluronic Acid)

**Figure 1:**
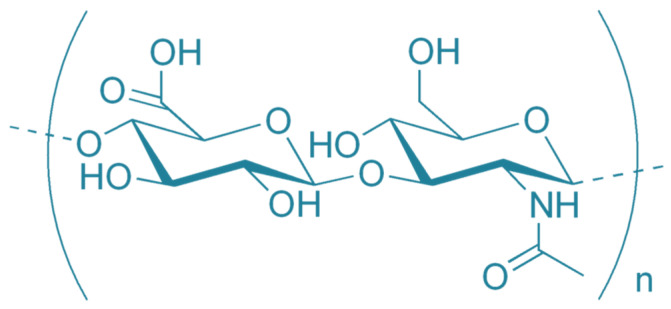
Structure of hyaluronan (HA).

HA (Figure 1), an abundant component of ECM, is a non-sulphated glycosaminoglycan consisting of N-acetyl-D-glucosamine (GlcNAc) and D-glucuronic acid (GlcUA) bound together by β1,3 and β1,4 glycosidic bonds. Repetition of this disaccharide leads to the formation of a long linear polymer with a molecular weight (MW) of several MDa. HA is not covalently linked to the core protein which makes it different from other glycosaminoglycans [17]. Due to its chemical structure and molecular size, HA is extremely hydrophilic and retains tissue hydration while acting as a passive space filler [18].

### 3.1. Metabolism of HA

#### 3.1.1. HA Synthesis

HA is synthesised from the cytosolic pool of UDP-glucuronic acid (UDP-GlcUA) and UDP-N-acetyl-D-glucosamine (UDP-GlcNAc) by three different isoforms of transmembrane HA synthases (HAS1, 2, 3) which release HA directly to the cellular environment [17]. HAS1 and HAS2 are responsible for the synthesis of high MW chains while HAS3 catalyzes the formation of molecules with lower MW (around 2 MDa vs. hundreds of kDa, respectively) [19]. Concerning enzymatic activity, HAS3 is the most active isoform, on the other hand, the activity of HAS1 is the lowest as a result of lower affinity to substrates [20]. However, the synthesis of HA by cells is affected by the availability of substrates and expression of HAS isoforms at the time. Growth factors and cytokines regulate the expression of HASs and, as a result, the synthesis of HA [21]. The importance of a particular HAS isoform is documented by studies employing HAS knockout mice revealing that constitutive ablation of HAS2 is embryonically lethal, while consequences of deletion of HAS1 or HAS3 are not as severe as in the case of HAS2 [22]. 

#### 3.1.2. HA Degradation 

Cleavage and degradation of HA are primarily mediated by hyaluronidases (HYALs) that are combined with degradation by other polysaccharide degrading enzymes as well as non-enzymatic degradation by free radicals. Six different HYALs are described to be present in humans. The most important and best described are HYAL1 and HYAL2 with enzymatic pH optimum at acidic range (3–4) and PH-20 with a pH optimum at neutral range (5–7). PH-20, based on the primary site of expression, also called testicular HYAL, is the most active one and is necessary for the process of fertilization [23]. While lysosomal HYAL1 is able to cleave HA chains with different MW and produces mainly tetrasaccharides in lysosomes, HYAL2 is mainly anchored in the cytoplasmic membrane and generates products with MW about 20 kDa (or approximately 50 disaccharide units) on the cell surface. The process of enzymatic HA cleavage is tightly connected with its receptor CD44 which is involved in the HA uptake by cells and is co-localized with HYAL2 [24]. HA cleavage is happening intensely in the so-called lipid rafts, special membrane structures which are formed by invaginations and differ from the membrane in their lipid and protein content. Acidification of lipid rafts is a necessary condition for the activation of HYAL2. Activated HYAL2 is then able to cleave HA to smaller fragments which are internalized to endosomes and transported to lysosomes where HYAL1 continues in their cutting [24]. Except HYALs, lysosomal β-glucuronidase and β-N-acetylglucosaminidase participate in HA catabolism and degrade HA fragments to monosaccharides, entering again to cellular metabolism [25]. Some authors speculate that the whole described process of HA degradation does not occur at the single-cell level. It seems that it could be divided between different compartments of the organism, for example, the initial cleavage could be provided in lymphatic or peripheral tissues, while the liver could be a place for the final degradation to monosaccharides. Interestingly, recent studies have proved the existence of other proteins with the ability to cleave HA including KIAA1199, also independently found and named as cell migration inducing protein (CEMIP), and transmembrane protein 2 (TMEM2, recently renamed to CEMIP2). The latter has probably great importance in the initial cleavage of HA on the cellular surface [26] but also in overall systemic catabolism and turnover of HA [27].

Besides the enzymatic HA degradation, HA can be cleaved by reactive oxygen species (e.g., superoxide anion, hydrogen peroxide, and hydroxyl radical) producing HA fragments with non-defined MW and changed the structure of the HA polymer [28].

### 3.2. Role of HA in Its Physiological Functions

HA produced by HAS1 and HAS2 is one of the largest molecules in the human body. Because of its simple chemical primary structure, its functions are mostly dependent on its size and in less extent also on covalent modifications.

#### 3.2.1. HA Fragmentation and Changes in Biological Properties

During normal HA turnover, HA fragments with different MW are formed. But those fragments are probably quickly metabolized intracellularly or eliminated from the organism. A higher concentration of polydisperse HA fragments can appear during inflammation [29]. These smaller HA fragments (of MW from 10^2^ to 10^5^ Da) are assumed, in contrast to high MW HA (HMW-HA; MW bigger than 10^5^ Da), to be pro-inflammatory by enhancing the production of pro-inflammatory cytokines and growth factors which are also responsible for angiogenesis and fibrotic related processes [30,31]. However, there also exist a few studies which did not confirm that low MW HA (LMW-HA) is pro-inflammatory as stimulation of immune cells was not observed after LMW-HA-treatment [32,33]. 

One mechanism of how HA fragmentation can affect its function is by loss of its mechanical and structural properties which are directly connected to MW of HA polymer. The extreme length of HA chains enables the forming of molecular meshworks at very low concentrations. This phenomenon is an important part of HA’s visco-elastic behavior in soft tissues such as vitreous humor and synovial fluid [34].

A decrease in molecular size can also affect the interaction of HA with other molecules. While chemically simple, HA is a very complex molecule from its interactome point of view. There are several classes of hyaladherins, i.e., molecules binding to the HA. Hyaladherins include components of ECM (hyalectans, link protein), soluble proteins (tumor necrosis factor-stimulated gene-6 protein, TSG-6; inter-α-inhibitor, IαI, a soluble form of CD44), HA cell surface receptors (CD44, LYVE-1, HARE, RHAMM, TLR-2/-4 or ICAM-1) and various other molecules including CEMIP. The most ubiquitous HA receptor is the CD44. It is present on almost all cells in the body and can exist in many isoforms due to large variability in alternative splicing. Signaling via CD44 affects inflammation, cell motility, tumorigenesis, injury resolution, etc., and has an important role in HA clearance and degradation. Lymphatic vessel endothelial HA receptor 1 (LYVE-1) is structurally close to CD44 and affects the entering of immune cells to lymph nodes [22] but is also responsible for HA clearance from the lymph. HA receptor for endocytosis (HARE) is present on endothelial cells in the lymph node, spleen, and liver. HARE binds HA, heparin, and chondroitin sulfate and rapidly removes them from the bloodstream [35]. Another HA receptor CD168, also known as the receptor for HA-mediated motility (RHAMM), is responsible for cellular transformation, proliferation, and motility of different cell types. HA fragments are also able to interact with Toll-like receptors, particularly with TLR-2 and -4, which are responsible for cell activation of pro-inflammatory cytokines and chemokines [36].

**Figure 2:**
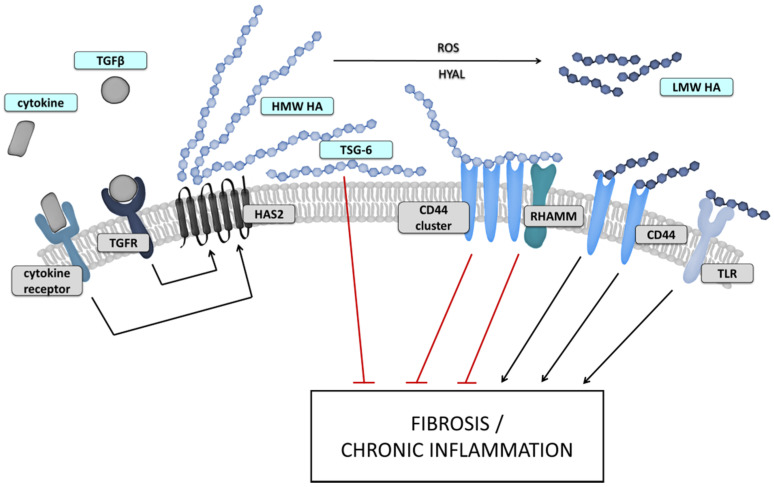
Postulated HA impact on peritoneal fibrosis. Cooperation between signals derived by TGFβ and other cytokine receptors affects HAS expression and activity. Produced HMW HA can be either cleaved by HYALs and ROS to smaller fragments or crosslinked, for example by TSG6. Subsequently, HA of different MW and HA complexes are able to influence pro-fibrotic conversion of MCs and connected inflammatory processes via receptor interaction or by interaction with other cell types.

The result of signaling pathways activated upon HA binding to receptors is dependent on the MW of HA chains (Figure 2) [17]. Generally, HA chains with HWM-HA are supposed to have protective properties for cells and tissues, on the other hand, fragments with lower MW are considered as signals of stress and initiators of defensive response [37]. This difference is best described in the HA/CD44 interaction. Surprisingly, HA binds to this receptor with relatively low affinity [38]. On the other hand, a single molecule of HMW-HA can bind to a large number of receptors, and HA’s MW also directly affect the reversibility of these bindings [39]. The multivalent and more stable binding of HMW-HA can further cause CD44 clustering which was not observed in short HA fragments [40]. But the exact mechanism of how cells are able to distinguish the LMW- and HMW-HA is still not fully explained as new research is challenging previous conclusions [41]. Moreover, the shortest fragments can probably bind only to some receptors. Nuclear magnetic resonance spectroscopy studies showed that the minimal oligomer able to bind to the CD44 is the hexasaccharide [38] while functional analysis suggests that TLR can bind even shorter HA fragments [42]. 

#### 3.2.2. HA Supramolecular Complexes

On the opposing spectrum of HA complexity are HA/protein aggregates which are formed, for example, under inflammatory conditions. TSG-6 and other hyaladherins, which are able to bind to multiple molecules, play a unique role in HA crosslinking and complex formation. In case of HA modification, TSG-6 first binds to heavy chains of IαI or pre-α-inhibitor (PαI) and catalyze covalent binding of these heavy chains onto HA molecules. Subsequent noncovalent interactions between heavy chains cause HA aggregation [43]. TSG-6/HA complexes can also interact with multivalent proteins pentraxin or thrombospondin creating stable HA/protein complexes. Last, TSG-6 enables a direct crosslinking of HA chains to form elongated HA fibers through the self-association of the TSG-6 protein [44]. These HA modifications and crosslinking are suggested to be involved in the regulation of physiological (oocyte cumulus matrix expansion) as well as pathophysiological processes (arthritis, pulmonary infections, autoimmune diseases, or scarring) [45,46,47,48,49]. In their review, Day and de la Motte propose the hypothesis that HA crosslinking serves as a protective mechanism. They propose that a dense structure of crosslinked HA matrix attracts the adhesion of leukocytes and thus limits immune cells binding to inflammation-promoting receptors on the underlying tissues [50]. 

Versican is another hyaladherin involved in ECM reorganization during inflammation. This proteoglycan binds to HA but also TSG-6 and IαI and together forms the so-called HA cables. These are HA-rich structures suggested to act as landing strips for leukocytes. Versican also sequesters various growth factors and cytokines [51] and interacts with various cellular receptors [52]. Most importantly, versican and HA create the so-called provisional matrix, a loose ECM that is present during the first stages of tissue repair and development which serves as a scaffold for immune and mesenchymal cells [53]. 

Based on this information, it is probable that the active biological role of HA is highly contextual. While its concentration, MW, and quaternary structure might direct its interactions and signaling pathways, the final outcome will be more dependent on the conditions in the surrounding tissues, including the degree of inflammation. Therefore, in the view of the fact that inflammation is one of the crucial factors participating in PA development, the above-mentioned information postulates that the level and structure of HA represents, together with the immune cells and pro-inflammatory mediators, another element interconnecting excessive inflammatory response with pro-fibrotic changes in the organism. 

### 3.3. HA and Energetic Metabolism

**Figure 3:**
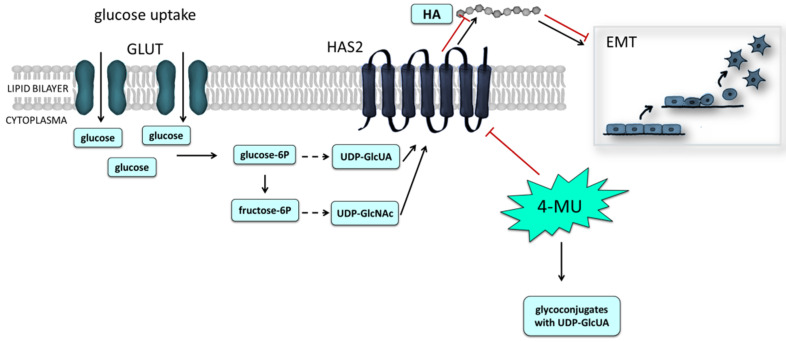
Connection of energetic metabolism and HA synthesis in activated MCs. Enhanced glucose uptake in metabolic active MCs leads to increased glycolysis resulting in higher accumulation of HA precursors (UDP-GlcUA and UDP-GlcNAc) and subsequently elevated biosynthesis of HA. Production of HA and its biological consequences (e.g., effect on endothelial-to-mesenchymal transition—EMT) can be inhibited by natural coumarin 4-MU.

Matrix remodeling in combination with other factors is connected with the regulation of cell metabolism. Concerning HA, an interaction of HMW-HA with a cell surface can shift cell metabolism towards glycolysis that is associated with endothelial-to-mesenchymal transition (EMT, a prototype of MMT) [54,55,56,57]. At the same time, an increased level of glycolysis in cells is necessary for the HA production that is dependent on glucose intermediates. Glucose-6-phosphate and fructose-6-phosphate arising during glycolysis are key intermediates of glycolysis which branch off from the main glycolytic pathway that serve as precursors for activated sugars, concretely UDP-GlcUA and UDP-GlcNAc (Figure 3). UDP-GlcNAc is synthetized through the so-called hexosamine biosynthetic pathway (HBP), a metabolic pathway derived from glycolysis [57,58]. Upregulation of HBP is implicated in the support of the cell growth and survival of HA-overproducing cancer cells [57,59]. In cancer and highly metabolically active cells, cellular metabolism is modulated. If glucose flux to oxidative phosphorylation and tricarboxylic acid cycle is blocked, glycolytic intermediates accumulate in the cells (Warburg effect). These intermediates, including the UDP sugars, create a source for various components essential in rapid cell proliferation. In addition to increasing HA synthesis, UDP-sugars stimulate binding of GlcNAc to certain serine or threonine residues of HAS2 and HAS3 (O-GlcNAc modification). This modification increases the lifetime of the enzymes, and their trafficking to the plasma membrane, where they are activated. The cellular content of UDP-GlcNAc and O-GlcNAc modification also control HAS2 transcription [59].

Because the HBP is interconnected with glycolysis, glycolysis is thus also influenced by the increased HBP flux. Cells respond to elevated consumption of UDP-sugars by increased glycolysis to preserve homeostasis. Chanmee et al. proved the existence of a metabolic switch toward glycolysis in HAS2-overexpressing cancer cells based on higher expression of enzymes involved in glycolysis and pyruvate dehydrogenase kinase 1, the enzyme which blocks the entrance of pyruvate, or more precisely acetyl-CoA, to tricarboxylic acid cycle [57,59]. High glucose uptake or over-expression of HBP-related enzymes promote the synthesis of HA that has an effect on cell physiology triggering cell growth and EMT as was primarily observed in tumor cell models. However, the interconnection between glucose metabolism and HA synthesis is apparent not only in cancer cells [57,58,59]. Sullivan et al. showed that an HYAL treatment increases glycolysis including a rise of glycolytic metabolites, glucose uptake, and lactate production in various cell types including primary and immortalized cells, as well as cancer cells. Correspondingly, an opposite effect was observed with apigenin, HYAL inhibitor, and inhibitor of HA synthesis, 4-methylumbelliferone (4-MU) [54]. 

Coumarins are currently the most recognized and experimentally employed inhibitors of HA production. 4-MU is a natural coumarin compound of plant origin, which can via reduction of the availability of HAS substrate UDP-GlcUA mediate the inhibition of HA synthesis in mammalian cells [60]. As well as the detailed effect on MCs, the exact mechanism of action of 4-MU on other cell types is unknown, but reducing the levels of UDP-hexoses, HA precursors, may alter the cellular phenotype and energy metabolism [61]. The connection between glycolysis and metabolism of HA was also studied in brown adipose tissue and chondrocytes with opposing results [58,62]. In brown adipose tissue, 4-MU increased respiration due to increased levels of glycolysis metabolites [58], whereas in chondrocytes, 4-MU reduced glycolysis and increased mitochondrial respiration related to enhanced phospho-AMP–activated protein kinase levels [62]. Interestingly, 4-MU reveals potent pharmacological effects. Inhibition of HA synthesis by 4-MU can significantly reduce the development of liver fibrosis, as it can affect cellular metabolism through inhibition of ECM synthesis, including HA [58,62,63].

## 4. HA in Peritoneal Fibrosis

### 4.1. Role of Endogenous HA in Peritoneal Fibrosis

Most studies related to the regulatory role of HA in the formation of PA are focused on a description of the application of materials from HA and its derivatives which are frequently used as components of antiadhesive barriers. Exogenic HA is there determined as an agent contributing to proper wound healing without the formation of fibrotic tissue [1,6]. Interestingly, the role of surgery-induced changes in HA synthesis and related metabolic changes in cells of peritoneal membrane in the development of postsurgical fibrosis and PA have not been studied yet. However, there are studies elucidating the involvement of endogenous HA in different inflammation and fibrosis-related conditions in the peritoneal membrane and abdominal cavity, processes connected to PA formation. 

MCs expressing typical markers as vimentin, cytokeratins, calretinin, mesothelin, and podoplanin line the peritoneal membrane and play a crucial role in pro-fibrotic processes in the peritoneum, synthesizing extracellular matrix related molecules, and a highly hydrophilic protective barrier, “glycocalyx”, consisting mainly of glycosaminoglycans including HA [64,65,66,67,68]. It is already well described that pro-fibrotic activation of MCs and peritoneal fibroblasts is reciprocally interconnected with inflammatory processes in the peritoneum [6,69]. Moreover, these processes are connected also to endogenous HA production. MCs modulate inflammation by synthesis and release of HA, which is able to sequester free radicals and initiate tissue repair responses [5]. Further, if HA and other extracellular matrix components constitutively present in the extracellular space are modified by injuries, they can become damage-associated molecular patterns—immune-stimulatory molecular patterns that can induce inflammation in case of sterile injury, recognized by CD44 receptor and others [4,5]. Moreover, failure to remove HA degradative products from sites of injury results in unremitting inflammation [31]. Numbers of neutrophils, macrophages, and other subtypes of peritoneal leukocytes and spectrum by them secreted soluble mediators is changed during PA development, resulting in up-regulation of pro-fibrotic activation of MCs. The amount of phagocyte derived reactive species cleaving HA molecules is enhanced during inflammation. In vitro, co-cultures of human peritoneal MCs with peritoneal macrophage-conditioned medium showed increased HA synthesis by the former cells [70]. These facts suggest that endogenously produced HA represents one of the regulators of intercellular communication between cells (e.g., MCs, fibroblasts, and different subsets of leukocytes) participating in PA development.

**Figure 4:**
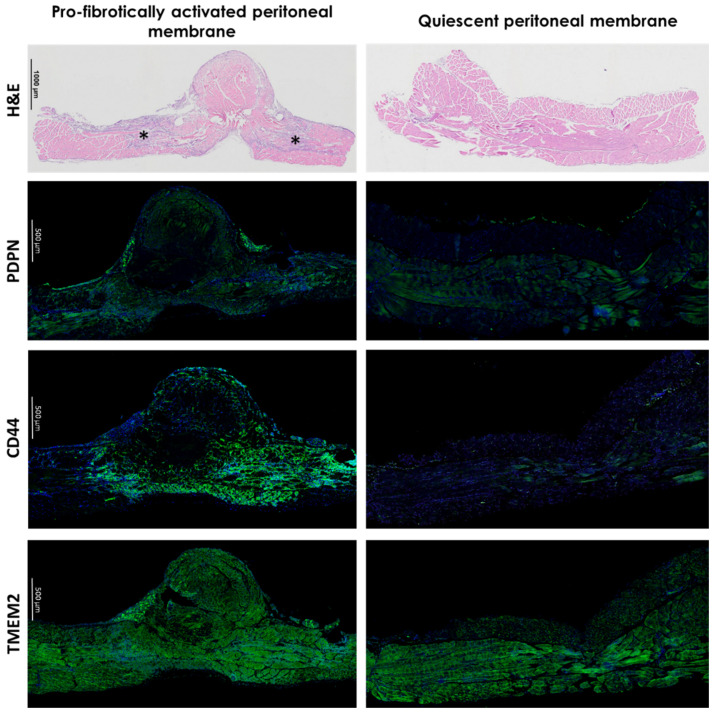
HA signal transduction is involved in PA development. Peritoneal membrane from mouse PA model 3 days after induction. In H&E images, fibrotic section was apparent (*). H&E-stained samples (DiaPath, Bergamo, Italy) were imaged by a light microscope (Nikon Eclipse 50i, Tokio, Japan) with a Plan Apo 4x/0.2 Nikon JAPAN objective. Images were processed using NIS elements software (Nikon, Tokio, Japan). Nuclei of cells are stained purple, extracellular matrix and cytoplasm are stained pink. Immunofluorescent staining revealed enhanced expression pattern for HA receptor CD44 (Abcam, #ab157107, 1/500, Cambridge, GB) in profibrotically activated peritoneal membrane, while expression of typical MCs marker podoplanin (PDPN, Abcam, #ab11936, 1/100, Cambridge, GB) and hyaluronidase TMEM2 (Invitrogen, #PA5-85901, 1/100, Waltham, Massachusetts, USA) in both quiescent and pro-fibrotically activated peritoneal membranes remained homogenous. Nuclei of cells were stained using ProLong™ Diamond Antifade Mountant with DAPI (Invitrogen, #P36962, Waltham, MA, USA). Peritoneal membrane was imaged using a laser scanning confocal microscope (Leica TCS SP8X, Wetzlar, Germany) with the Leica HC PL APO CS2 20x/0.75 IMM (Zoom 1) objective. Images were processed using Leica LasX software and are representative of three mice. Abbreviations: PA: peritoneal adhesions, H&E: Hematoxylin and Eosin, PDPN: podoplanin, DAPI: 40,6-diamidino-2-phenylindole.

The illustration of the proposed importance of HA in PA is provided in Figure 4. showing a representative IHF staining for typical MCs marker podoplanin and HA receptor CD44 and hyaluronidase TMEM2 in a mouse model of induced PA.

The modulation of HA metabolism after a mechanical injury of human peritoneal MCs in vitro was previously described by Yung et al. Up to 24h after an injury to the mesothelial cell monolayer, up-regulation of HAS2 and down-regulation of HAS3 expression was observed. During the same period, HA production was significantly increased. Further, in additional experiments, a dose-dependent effect of HA on cell migration and wound closure was observed [71]. Endogenous production of HA also regulated angiogenesis in the peritoneum [72]. These data were in agreement with in vivo observation of microvillus-like cell protrusions on rat mesothelium positive for HA and HAS1–3 staining [73].

As the interconnection of HA metabolism and inflammatory response was described, HA fragments markedly enhanced the mRNA expression and protein synthesis of chemokines MCP-1 and IL-8 in cultured human peritoneal MCs [74]. Pro-inflammatory IL-1β is a key cytokine controlling HA synthesis in the mesothelium and is over-produced during peritoneal dialysis (PD), a therapeutic procedure often connected with inflammation and fibrosis of the peritoneal membrane [75]. It is known that higher levels of pro-inflammatory mediators and HA occur in the eluent of patients who undergo PD. Raby et al. described a TLR4 mediated activation of peritoneal MCs and subsequent fibrosis in the context of PD. Exposure of peritoneal cells (leukocytes and MCs) to PD solution yields increases in Hsp60, Hsp70, and HA which interact with TLR2 and TLR4 receptors on peritoneal cells and trigger inflammatory processes via ERK1/2 phosphorylation leading to fibrosis [76]. Further, de la Motte et al. reviewed, how the endogenous HA can modulate intestinal fibrosis via inflammatory processes in the abdominal cavity. She suggested a key role of HA binding to CD44 on macrophages, clearing the inflammatory HA matrix and resolving inflammation during the progression of colitis. In PA development, the role of leukocytes has also been described, however, participation of HA in this process is anticipated, but not exactly proven [3,77,78]. The role of CD44 was studied also in endometriosis, another pathological condition connected to inflammation in the peritoneal cavity, which can result in fibrosis and PA formation. During endometriosis small fragments of the endometrium are released into the peritoneal cavity, they can attach to the mesothelial monolayer and additionally grow and thus cause inflammation and PA formation. The role of HA in endometriosis was studied by Dechaud et al. They proved that pretreatment of MCs with HYAL reduced attachment of endometrial stromal and epithelial cells to the mesothelial monolayer. Pretreatment of endometrial cells had no effect on adhesions suggesting the importance of MCs released HA [79]. A later study was focused on the determination of the role of CD44 in the attachment of fragments of the endometrium to MCs. Inhibition of N- and O-linked glycosylation of CD44 on endometrial cells led to a significant reduction in adhesion to mesothelial monolayer suggesting the importance of CD44 in adhesion to the mesothelial monolayer [80]. 

Maintaining the equilibrium between fibrin-dissolving and fibrin-forming systems (i.e., fibrinolysis and coagulation respectively) is crucial for normal peritoneal tissue repair avoiding PA formation. Effect of HA fragments is suggested also in fibrinolysis, as the increased expression of plasminogen activator inhibitor-1 by HA fragments was observed during peritoneal dialysis [81].

Moreover, there are indirect proofs that HA structure and HA interactions with multiple molecules might be important players in PA formation. Wang et al. studied the role of TSG-6, the key protein responsible for HA crosslinking and HA complex formation in a reduction of PA development. Their results indicated that rat bone marrow-derived mesenchymal stem cells may attenuate peritoneal injury by repairing MCs, reducing inflammation and fibrosis. Rather than engraftment, the secretion of TSG-6 was proved to have a major contribution to the therapeutic benefits of mesenchymal stem cells [82]. Similarly, other HA binding proteins, pentraxin and thrombospondin, just like versican, are implicated in the development of peritoneal fibrosis [83,84,85].

### 4.2. Cues from Other Tissues

Looking at other tissues and organs than the peritoneum, there exist significantly more studies evaluating the role of HA in fibrosis. Most of these studies focus on pro-fibrotic effects of transforming growth factor β (TGFβ) that is connected with activation of HAS2 and higher production of HA in fibrosis-related pathologies in different tissues. However, also physiological upregulation of HAS2 and related higher production of HA was described during follicular development and ovulation in human TGFβ activated granulosa-lutein cells through SMAD2/SMAD3-SMAD4 pathway [86]. Further, it was recently shown that, for example, atrial fibroblasts treated by TGFβ produce higher levels of HA in vitro. Moreover, HA was upregulated in atria of TGFβ transgenic mice and patients with atrial fibrosis [87]. Accumulation of HA was observed also in the case of renal fibrosis. Detection of HA turnover revealed that on the first day after injury mainly HMW-HA is accumulated. On the contrary, two weeks later, medium, and low molecular fragments are present. This observation correlates with up/downregulation of HASes and HYALs in time [88]. However, it seems that the production of HA in fibrotic conditions is not only a passive consequence of cell activation but HA can also affect processes related to the TGFβ signaling pathway, as was observed for example in cardiac fibroblasts [89]. Reduction of αSMA expression was observed after inhibition of HA synthesis [90]. The same was shown also in the case of renal fibrosis, moreover, suppression of macrophages and neutrophil accumulation and collagen deposition was detected [91]. Meran et al. found that TGFβ treatment of dermal and oral fibroblasts (models of scarring and nonscarring phenotype) caused an increase of HAS2 expression and myofibroblast differentiation only in the case of dermal fibroblasts. Inhibition of HA production in dermal fibroblasts led to their failure to differentiate and SMAD signaling was attenuated [92,93]. Similar to dermal fibroblasts, overexpression of HAS2 in lung fibroblasts supported their activation and differentiation to myofibroblasts. The importance of HAS2 in the development of lung fibrosis was confirmed on the murine in vivo model of bleomycin-induced injury by using conditional HAS2-KO mice which did not suffer from fibrosis after bleomycin treatment. Jenkins et al. described for the first time a change in HA turnover as a result of the differentiation of lung fibroblasts to myofibroblasts and highlighted the potential importance of the HYAL enzymes in controlling fibrotic progression. HA accumulation changed during the activation of fibroblasts and was a major contributor to the maintenance of the cells in that phenotype. The mentioned study suggested that the greater accumulation of HA by myofibroblasts may be more likely the result of decreased HA degradation than enhanced HA synthesis [94].

The role of HA in lung fibrosis is nowadays very topical also in the context of pandemic viral pneumonia—coronavirus disease 2019 (COVID-19). Upon infection with the severe acute respiratory syndrome coronavirus (SARS-CoV2), HAS2 expression is excessively elevated in the consequence of overproduced pro-inflammatory cytokines (e.g., IL-1β and TNF-α), which leads to enhanced production of HA (the so-called “HA storm”). On one hand, overproduced HMW-HA can absorb water and contribute to lung edema and negatively affect lung function, on the other hand, HA fragmentation and LMW-HA formation are associated with the production of pro-inflammatory mediators and might deepen lung inflammation, remodeling, and fibrosis [95,96]. 

Mechanisms of HA contribution to fibrosis-related processes remain to be fully clarified, but the involvement of HA receptors is presumed. Li et al. observed the involvement of CD44 in the development of lung fibrosis in mice since bleomycin-induced injury was inhibited by blocking antibodies to CD44. The invasive phenotype of fibroblasts isolated from patients with idiopathic pulmonary fibrosis was HAS2 and CD44 related [97]. The role of CD44 was also determined in the study of Muller et al., which focused on the involvement of CD44 in post-infarctional inflammation. The level of pro-inflammatory cytokines including CCL5 and MCP1 in media of IL-6 treated cardiac fibroblasts was diminished by CD44 deletion [90]. CD44 interactions may play an important role in the resolution of inflammation and the migration of fibroblasts in injured tissues as Huebener 2008 showed on a model of cardiac remodeling. However, it is important to mention that the diversity of CD44 biological activity is probably dependent on the generation of distinct CD44 isoforms through alternative splicing [98]. Midgley et al. (2017) identified an unexpected function for HYAL2, as a regulator of CD44 splicing [99].

The role of CD44 was also described in liver inflammation and fibrosis. CD44-KO mice showed a significant decrease in the expansion of inflammation and fibrosis [100]. Results from a recently published study by Yang et al. confirmed the importance of CD44 in liver fibrosis and also showed the role of receptor TLR4. They suggested that fibrogenesis of hepatic stellate cells is driven through activation of Notch1 as a consequence of LMW-HA, CD44, and TLR4 interaction [101]. 

Another suggested mechanism of HA-action is due to increased activation of macrophages via RHAMM receptor which can lead to prolonged inflammation and finally to fibrosis. The role of RHAMM was studied in lung fibrosis on a model of bleomycin-induced injury. Increased expression of RHAMM and higher motility were observed on isolated alveolar macrophages from bleomycin-treated rats in contrast to macrophages treated by compound R36 which is known as an inhibitor of RHAMM [102]. The importance of RHAMM was recently confirmed by Cui et al. employing a similar model. They compared the development of inflammation and fibrosis in the lungs of wild-type mice, RHAMM-KO mice, and mice with overexpression of RHAMM in macrophages after bleomycin treatment. As they supposed, the RHAMM-KO mice had reduced fibrosis in contrast to the wild-type mice, on the other hand, the transgenic mice suffered from the most extensive fibrosis [103].

**Figure 5:**
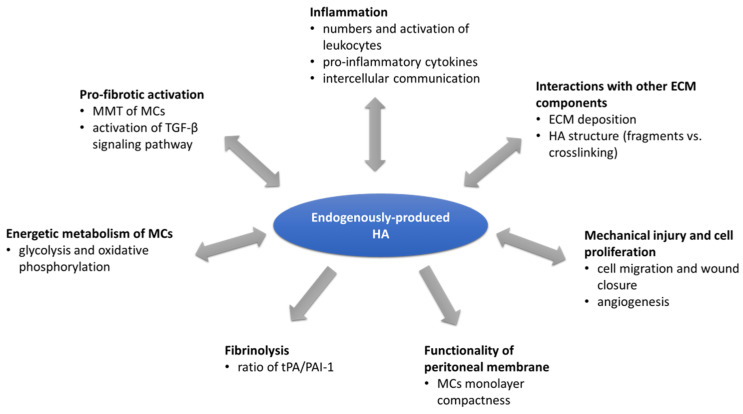
Endogenous production of HA is interconnected with processes participating in peritoneal fibrosis and regulation of PA development.

An overview of processes interconnecting endogenous production of HA with PA development is summarized in Figure 5.

A limitation of most studies evaluating the effects of exogenously applied HA on fibrosis is the missing information about MW of HA used. Further, the action of HYALs and related HA chain fragmentation and degradation are probably other factors affecting fibrosis that are not considered in the above-mentioned studies. An exception is a study of Colombaro et al. who showed on the HYAL1/2-KO murine model of renal ischemia-reperfusion injury that the lack of either HYAL1 or HYAL2 and accumulation of HA leads to exacerbated inflammation and fibrosis accompanied by increased expression of HA, CD44, α-Smooth muscle actin (αSMA) and collagen I and III [104]. 

### 4.3. Anti-Fibrotic Effects of Endogenous HA

Except for the before-mentioned papers describing the stimulatory effect of enhanced HA production and signaling on fibrosis, there also exist studies showing a beneficial action of endogenously produced HA on fibrosis-related conditions. During peritoneal inflammation accompanying PA development, increased HA synthesis provides a protective signal to MCs and fibroblasts to promote tissue survival and repair by the formation of intercellular cables preventing peritoneal leukocytes activation and interaction with inflammation-promoting receptors [31]. Further, Evanko et al. showed the importance of endogenously produced HA in pericellular matrix of fibroblasts. Inhibition of HA production by 4-MU contributed to myofibroblast phenotype, higher deposition of fibronectin, collagen I, and increased expression of αSMA and HAS2 were observed [105]. Recently, Wang et al. proved on the model of renal injury that the anti-fibrotic effect of cytokine IL-10 is driven through HMW-HA production by upregulated HAS2 dependent on STAT3 activation [86]. On the other hand, an earlier study of renal injury suggested that inhibition of HA receptor CD44 by oligo-fucoidan reduced fibrosis, and additional HA diminished this anti-fibrotic effect in vitro. Moreover, a decrease of HA after oligo-fucoidan treatment was observed in the in vivo model [106]. These opposite results suggest that the HA action is not different across tissues but that it is probably driven by a very complex machinery of mechanisms and conditions, including, e.g., ratio of HA fragments with different MW, and that must be solved as comprehensively as possible.

## 5. Conclusions

As we showed in this review, endogenous production of HA in the peritoneum can affect most processes participating in peritoneal fibrosis and regulation of PA development and vice versa (Figure 5). Importantly, HA regulates the action of cells participating in PA development, e.g., MCs, fibroblasts, and different subsets of leukocytes. Inhibition of HA production from MCs can verifiably influence the transformation of non-activated cells to myofibroblast phenotype, in which case the switch of cell metabolism towards glycolysis is the crucial accompanying feature. On the contrary, a key pro-fibrotic mediator TGFβ can upregulate the expression of HAS in MCs and peritoneal fibroblasts. Therefore, it is evident that HA synthesis and fibrosis are interdependent processes. Moreover, the indirect effect of HA via regulation of inflammation as well as cell proliferation is of fundamental importance in peritoneal adhesions formation as observed in fibroblasts, chondrocytes, brown adipose tissue, cancer cells, etc. Therefore, the unrevealing of exact mechanisms of regulation of pro-fibrotic processes by endogenous production of HA in the context of the peritoneum is essential for understanding the formation of PA. Currently, there are new tools that can help us to investigate the role of endogenous HA in PA more deeply, including HAS and HYAL knockouts, which can significantly help us to extend the knowledge of this topic and also to implement effective strategies to prevent these pathological processes.
